# Effect of Weight Self-Stigma and Self-Esteem on Aerobic Exercise Capacity in Adult Women with Different Body Compositions

**DOI:** 10.3390/ijerph19020873

**Published:** 2022-01-13

**Authors:** Monira I. Aldhahi, Wafa K. Al Khalil, Rawan B. Almutiri, Mada M. Alyousefi, Bayader S. Alharkan, Haya AnNasban

**Affiliations:** Department of Rehabilitation Sciences, College of Health and Rehabilitation Sciences, Princess Nourah bint Abdulrahman University, P.O. Box 84428, Riyadh 11671, Saudi Arabia; WafaKhalil0@gmail.com (W.K.A.K.); rawan.bader95@gmail.com (R.B.A.); mada1418@gmail.com (M.M.A.); Bayaderalharkan@gmail.com (B.S.A.); hayanasban@gmail.com (H.A.)

**Keywords:** cardiopulmonary fitness capacity, self-esteem, weight self-stigma, obesity, overweight, body composition

## Abstract

Background: Overweight individuals face weight-related stigmatization, driving self-exclusion from exercise and physical activity. The extent to which weight self-stigma and self-esteem are associated with aerobic capacity remains unclear. Therefore, this study aimed to characterize the cardiopulmonary fitness, weight self-stigma, and self-esteem of overweight women and examine whether weight self-stigma and self-esteem predict cardiopulmonary aerobic capacity. Methods: A cross-sectional study was conducted with 66 women stratified into two groups: a normal weight (NW) group and an overweight (OW) group. The mean body mass indexes and ages of the NW and OW groups were 20.4 ± 0.36 kg/m^2^ and 29.5 ± 0.8 kg/m^2^, and 24 ± 7 years and 21 ± 3 years, respectively. Submaximal exercise testing using the modified Bruce treadmill protocol was conducted to measure the predicted oxygen uptake (VO_2 peak_) and energy expenditure. The Weight Self-Stigma Questionnaire and the Rosenberg Self-Esteem Scale were used. Results: Significantly lower mean of predicted VO_2 peak_ and higher mean of energy expenditure were reported in the OW group compared with the NW group (25.8 ± 5.3 mL/kg/min vs. 28.7 ± 4.8 mL/kg/min, *p* = 0.001 and 9.7 ± 1.9 kcal/min vs. 7.5 ± 1.8 kcal, *p* = 0.03, respectively). There was a significant difference in weight self-stigma and self-esteem between the groups. Regression model analysis indicated that weight self-stigma and self-esteem explained 45% of the variance in the predicted VO_2 peak_. Conclusion: Strategies enhancing self-esteem and avoiding stigmatization should be embraced to promote fitness and engagement in physical activity among OW women.

## 1. Introduction

Functional aerobic capacity (FAC) is important for the maintenance of cardiovascular function and has been shown to play a protective role in reducing the risk of metabolic disorders [1], heart disease [2], and even mental health [3]. Maximal oxygen uptake (VO_2max_) which is an indicator of an adequate level of FAC, reflects the sufficient amount of oxygen relative to metabolic demand needed to sustain prolonged exercise [4]. The amount of VO_2max_ during incremental exercise has been recognized by the World Health Organization as the best determinant of cardiorespiratory fitness [4]. It is a strong and independent predictor of cardiovascular health, mortality, and morbidity in adults [5]. The capability to perform exercise and activities daily is multifactorial and linked to an efficient function of central hemodynamics, muscle energy metabolism, and psychological health [6]. The importance of integrating a positive psychological perspective into health has been recognized in the International Classification of Functioning, Disability, and Health (ICF) model [7]. The ICF allows for an extensive understanding of several factors at the personal, psychological, and environmental levels that may negatively affect the activity performance and exercise capacity of obese individuals [8]. Obesity has become a major global concern, with the World Health Organization reporting that more than 1.9 billion adults are overweight (OW) and 650 million are considered obese [9]. The prevalence of obesity in Saudi Arabia is concerning, with reports of higher prevalence in women than men (33.5% vs. 24.1%, respectively) [10]. Worldwide, a high body fat composition is considered a dominant major public health concern. It is associated with many comorbidities, such as type 2 diabetes mellitus and coronary heart disease [11].

A high body fat composition can contribute to changes in aerobic capacity and psychological health. Previous studies have demonstrated a strong association between a higher body mass index (BMI) and reduced cardiopulmonary fitness [12,13]. From a physiological perspective, body composition and fitness influence physical performance. With body composition playing an important role in determining energy expenditure, obesity has been shown to significantly drive the increase in energy expenditure when compared with individuals with normal body weight (NW) [14]. These findings suggest that higher energy expenditure in obese individuals results from having greater amounts of fat tissue which leads to an increase in cardiorespiratory work. From a psychological perspective, an individual’s self-esteem and weight self-stigma form the foundations of human performance and motivation to accomplish tasks. Perceived self-esteem and awareness of body size may be reflected in their attitudes and physical performance. Previous studies have reported that obese individuals feel stigmatized about their body weight and have lower self-esteem than individuals with NW [15,16,17,18]. Additionally, weight-based stigmatization has been shown to have negative psychosocial outcomes, such as diminished desire to lose weight and decreased self-esteem [19,20]. The dual pathway between weight self-stigma and self-efficacy has been investigated and may direct an individual’s beliefs in their ability to function, hindering participation in physical activity [21]. A systematic review revealed that internalization of weight bias was associated with negative mental health outcomes, including self-esteem, depression, and anxiety [22]; this association, therefore, extends to the context of physical activity. Few previous studies have examined the association between weight bias internalization and physical health [23,24,25].

Literature focusing specifically on physical fitness, self-esteem, and weight bias internalization is sparse. Our current understanding of the predictors of FAC is limited, and in-depth research is needed to understand the impact of psychological factors such as self-esteem and weight self-stigma on fitness to promote better inclusion of OW women in physical activity. Understanding the psychological aspects of OW individuals is important for understanding the risks of sedentary behavior. Previous studies have focused on self-esteem and stigma in individuals with a high body fat composition, without exploring its impact on FAC. This study seeks to address two research questions: (1) Does body composition affect women’s self-esteem, weight stigma, and cardiopulmonary fitness? (2) To what extent are self-esteem and weight self-stigma associated with cardiopulmonary fitness among women with different body compositions? The overarching objectives of this study were two-fold. First, it aimed to characterize the differences in FAC, weight self-stigma, and self-esteem between an OW group and an NW group. The current study hypothesized that the FAC, weight self-stigma, and self-esteem may differ between women who are OW compared with NW women. The second objective was to assess the extent to which self-esteem and weight self-stigma influenced FAC among adult women with high body fat composition. It was hypothesized that weight self-stigma and self-esteem influence peak oxygen uptake, which is a proxy for FAC. Our study may contribute to uncovering the interplay between self-esteem, self-weight stigma, and FAC, the understanding of which could enhance health and quality of life in these subsets of the population. The findings of this study are intended to lay the groundwork for the physical health practitioners to promote inclusive cognitive-behavioral therapy among women with overweight to enhance physical fitness.

## 2. Materials and Methods

### 2.1. Study Design and Population

A cross-sectional study was conducted with 66 women Saudi participants between 18 and 45 years of age. The study was conducted at the functional performance lab at Princess Nourah bint Abdulrahman University. A nonprobability convenience sampling method was used to recruit participants and printed announcements were distributed to inform potential participants. Inclusion criteria specified women between the ages of 18 and 45 years old. Participants in the study had to be able to walk independently on a motorized treadmill. A BMI was specified to be >18.5 kg/m^2^ was required. Participants were excluded from the study if they had a history of cardiopulmonary impairment, neurological or musculoskeletal deficits, a history of obstructive lung disease, medication that would influence cardiovascular function, nicotine use, or pregnancy. Group categorization was based on body fat percentage (BF%) and BMI. The overweight/obese group included women with high %BF (>32%) and BMI between the range of 25 kg/m^2^ -29.9 kg/m^2^; the comparison group, included females with normal %BF (>21 to <32%) BMI < 24.9 kg/m^2^ [26].

### 2.2. Ethical Considerations

This study was approved by the Internal Review Board Committee of Princess Nourah bint Abdulrahman University (IRB #/21-0048). The study protocol, procedures, and rights were explained at the beginning of the study. Prior to participation, written informed consent was obtained from all participants.

### 2.3. Study Procedures

Inclusion criteria evaluation and screening for exercise appropriateness using the general health history questionnaire and Physical Activity Readiness Questionnaire plus (PARQ+) were performed. Before conducting the modified Bruce protocol for each participant, anthropometric measurements were taken. The anthropometric and blood pressure measurements, as well as the modified submaximal Bruce protocol, are described below.

#### 2.3.1. Anthropometric Assessment

A stature meter with a precision of 0.01 cm was used to measure height in centimeters using a standardized method. An electronic digital scale was used to measure weight to the nearest 0.1 kg. Subjects were asked to stand barefoot on the balance plate while their weight was measured. BMI (kg/m^2^), fat-free mass %, and BF% were measured using a smart scale. To measure the BF%, a bioelectrical impedance analysis was used. The participants were instructed to stand barefoot on the bioelectrical impedance analysis devices (seca mBCA 515, Hamburg, Germany), which were validated directly against the direct gold standard measurement [27]. The device measures the body composition and includes a platform with handrails and electrodes that measure the impedance passed through the pairs of hands and feet while standing. Impedance was measured over 75 s with a current of 100 μA at a frequency range between 1 kHz and 1000 kHz and a capacity of 360 kg. Waist circumference measurement at the umbilicus and level physical activity level were inputted to indirectly estimate the body composition. BMI was calculated as weight divided by height squared (kg/m^2^).

#### 2.3.2. Blood Pressure Measurements

Brachial blood pressure was measured manually at rest prior to the start of the exercise and during recovery at 1 min post-exercise and 6 min post-exercise. Heart rate was measured at rest and simultaneously during exercise using a Polar heart rate monitor [28].

#### 2.3.3. Bruce Submaximal Treadmill Protocol

**Figure 1:**
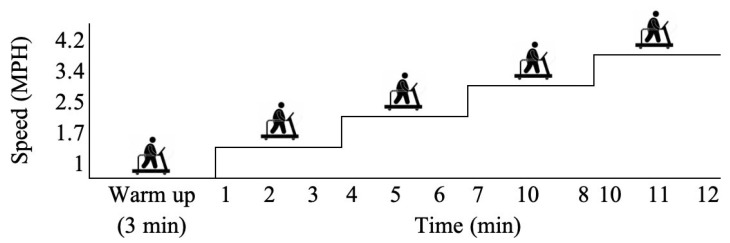
Modified Bruce submaximal treadmill protocol.

This protocol outlines a multistage treadmill test for assessing cardiopulmonary fitness. The test was administered in 3-min stages until volitional exhaustion, as reflected by 95% of the participant’s maximum heart rate. To ensure the participant’s safety and to monitor the volitional exhaustion point, the heart rate was measured simultaneously every 2 min using a Polar heart rate monitor (Polar Electro Inc., Bethpage, NY, USA). The criteria for termination of the motor-driven treadmill protocol were volitional exhaustion using the rate of perceived exertion (Borg Rating of Perceived Exertion [RPE] 6–20) and 95% of the age-predicted maximal heart rate. The test is typically performed with maximal effort to predict maximal oxygen uptake, which is reflected by oxygen consumption (VO_2 peak_ [mL/kg/min]) and the duration of the exercise [29,30]. The participants are instructed to walk on the treadmill using stepwise increases in speed and grade for 12 min. Following a 3 min warm-up at 1 mph and 0% grade, the speed and grade were increased by 2% increments every 3 min until volitional exhaustion (Figure 1) [31].

The predictive equation (Equation (1)), which has been validated for women, was used to calculate the VO_2 peak_ as follows:

(1) $${\mathrm{VO}}_{2\;\mathrm{peak}}=4.38\times \left(\mathrm{time}\right)-3.90\;$$

Energy expenditure is defined as the amount of energy utilized relative to metabolic demands, known as caloric cost. The operational formula (Equation (2)) is used to calculate energy expenditure:

(Energy expenditure = (Metabolic equivalents × 3.5 in mL/kg/min × body weight in kg)/(200 in kcal·mL^−1^)) (2)

where 200 kcal·mL^−1^ is 1000 mL·L^−1^ ÷ 5 kcal·L^−1^, unite kcal/min [32].

#### 2.3.4. Study Questionnaires

The Rosenberg Self-Esteem Scale (RSES) and Weight Self-Stigma Questionnaire (WSSQ) were used in this study and required approximately 10 min to complete.

Weight Self-Stigma Questionnaire (WSSQ): The questionnaire includes 12 items to assess the participant’s fear and judgments of others regarding their body shape and weight. The responses were ranked on a 5-point Likert-type scale ranging from 1 (“completely disagree”) to 5 (“completely agree”). The total score was the sum of all responses, which ranged between 12 and 60, and there were no reverse calculations [19]. A higher score indicated more shame related to body weight and shape. The composite score was the sum of all scale items. The WSSQ showed an acceptable internal consistency and reliability as Cronbach’s alpha was 0.78 [24].

Rosenberg Self-Esteem Scale (RSES): The questionnaire consists of 10 items that are self-administered and easy to use [33]. The main aim of this scale was to measure the participants’ self-esteem [34]. The responses were ranked on a 4-point Likert-type scale ranging from 1 (“strongly agree”) to 4 (“strongly disagree”). Items 2, 5, 6, 8, and 9 were reverse scored. The overall responses were integrated into a single, composite response that ranged between 10 and 40, where higher scores indicated higher self-esteem [33]. The RSES has been widely used in cross-cultural studies including a wide range of sample populations (e.g., men over 60, students, and civil servants) with alpha coefficients ranging from 0.77 to 0.88 [33,35,36]. The reported test-retest reliability after a 2-week interval was 0.85 [37].

International Physical Activity Questionnaire (IPAQ): All participants were questioned regarding their weekly physical activity status as determined using the 7-Day Physical Activity Recall questionnaire and a total duration of physical activity was determined. The IPAQ records the participant’s recall of their physical activity in the last 7 days and defines four levels of intensity of physical activity which are a vigorous-intensity activity, moderate-intensity activity, walking, and sitting. The IPAQ had a high internal consistency reliability with a Cronbach’s alpha of 0.76 [38].

### 2.4. Statistical Plan

#### 2.4.1. Study Sampling

This study investigated the association between FAC, as reflected by predicted VO_2 peak_, self-esteem, and weight self-stigma among OW women. The G*power version 3.0.10 (http://www.psycho.uni-duesseldorf.de/abteilungen/aap/gpower3/, accessed date 9 January 2022) estimate of the sample size was used and a statistical power consideration included an effect size (ES) of 0.35 with a margin of error at 5% and 95% power with a confidence index level set to 95%. The estimated sample size required for this study was 66 women. We established a recruitment target of at least 33 women for each group for recruitment.

#### 2.4.2. Statistical Analysis

We screened all data to detect the presence of any missing data, outliers, and normality. The Shapiro-Wilk test was performed to evaluate the normality assumption of the data. Data are presented as mean and standard deviation (SD) for continuous variables, and frequency and percentage (%) for categorical variables. Baseline data were analyzed using an independent *t*-test to compare the variables of interest between the groups. For the variables of interest, an analysis of covariance (ANCOVA) was used to assess the mean differences after adjusting for age. Pearson product-moment correlations were used to examine the relationships between self-esteem and weight self-stigma. A forward selection multiple linear regression analysis was then carried out to assess the association between the independent variables and predicted peak oxygen uptake. Changes in the R^2^ were compared in the models to understand the variance as explained by self-esteem and weight self-stigma in the predicted peak oxygen uptake. Statistical significance was set at *p* < 0.05. The collected data were analyzed using Stata version 16 (StataCorp, College Station, TX, USA).

## 3. Results

Overall, 66 healthy women were assigned to either the NW or OW group. The mean weights in the NW and OW groups were 52 ± 5.9 kg and 75.8 ± 9.9 kg. Table 1 shows the physical characteristics of the participants in this study. Both groups started with a similar baseline, and there were no significant differences between the two groups except for the differences in systolic blood pressure (BP) and age. As demonstrated in Table 1, there were significant differences between the two groups in BMI (kg/m^2^), fat mass (%), body weight (kg), and muscle mass (%).

**Figure 2:**
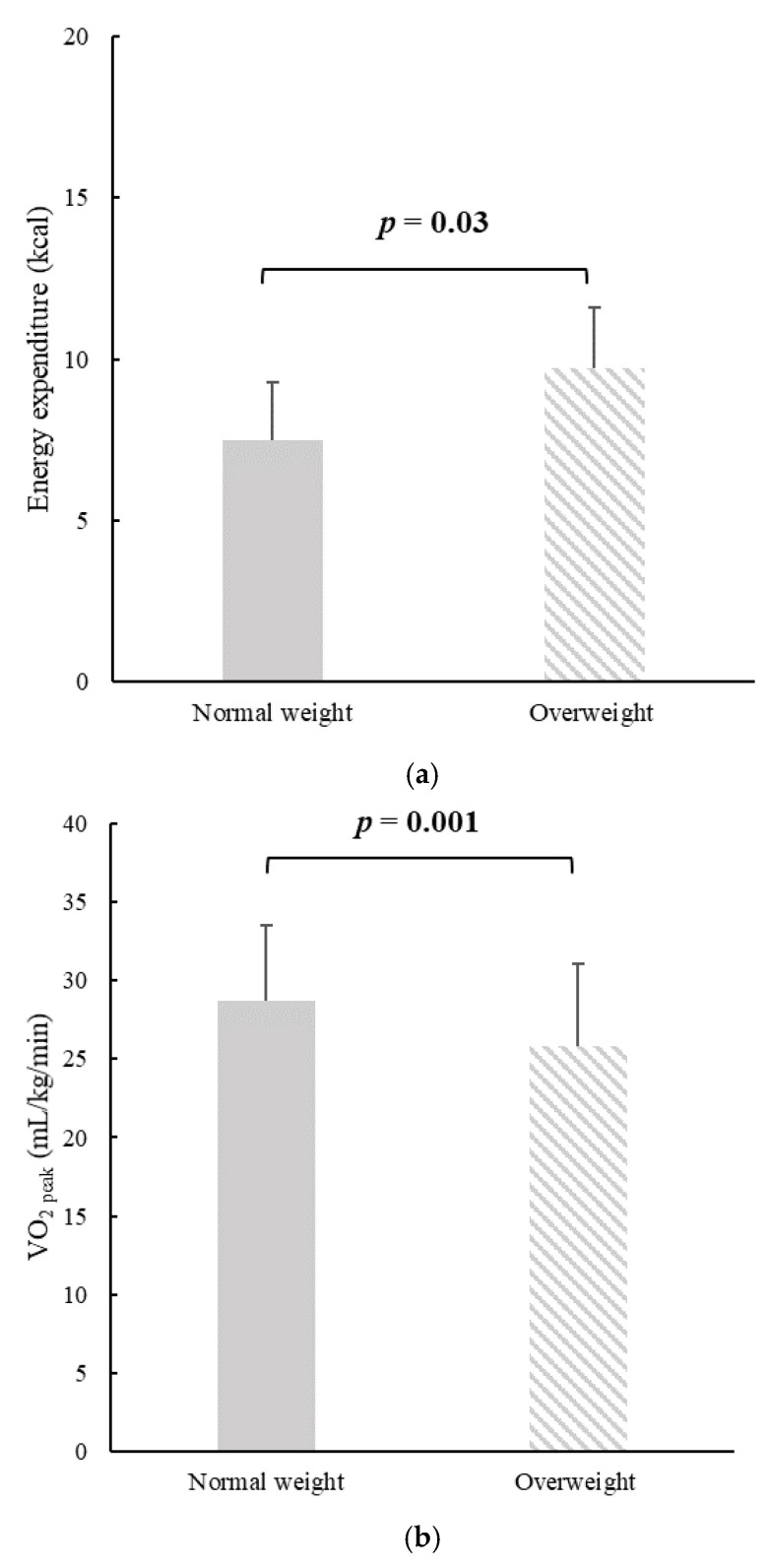
Comparison of mean (**a**) energy expenditure, (**b**) predicted oxygen uptake at peak of exercise between the study groups.

At peak exercise, diastolic BP and peak HR were not significantly different between the two groups. However, there was a difference between those with OW and NW in systolic BP at the peak of the exercise (OW: 130 ± 10 mmHg; NW: 127 ± 5 mmHg, *p* < 0.0001). Table 2 presents a comparison of FAC, self-esteem, and weight self-stigma between the two groups. The findings showed that there were significant differences between the two groups in VO_2 peak,_ self-esteem, and body weight self-stigma. Figure 2 illustrates the significant differences between the groups in VO_2 peak_ and energy expenditure. Higher oxygen consumption of 3 mL/kg/min was reported in the NW group, which is a proxy for fitness. Of particular interest, there was a significant difference in the exercise duration of approximately one minute between the NW and the OW groups.

Multiple regression analyses were used to test whether weight self-stigma and self-esteem significantly predicted peak VO_2_ (Table 3). The results of the regression indicated that weight self-stigma and self-esteem explained 45% of the variance in peak VO_2_ (F (5, 61) = 5.86, *p* = 0.001). Additionally, the Pearson correlation coefficient showed negative correlation between weight self-stigma and self-esteem (r = −0.35; *p* = 0.004).

## 4. Discussion

This study aimed to characterize the differences in FAC, self-esteem, and weight self-stigma among adult women between 18 and 45 years of age. We investigated the extent to which self-esteem and weight self-stigma were associated with FAC. The findings of the current study indicated a significant decrease in predicted VO_2 peak_, which reflected a lower FAC in the OW participants compared with the NW group. These findings are concurrent with decreased exercise duration among the OW group. Although the exercise duration was lower in the OW group, their energy expenditure was higher. The OW group demonstrated a significantly lower composite self-esteem score and higher weight self-stigma than the NW group. The high weight self-stigma score suggested weight-based stigmatization and body dissatisfaction among group members, which could have a deleterious effect on exercise capacity. In support of this, we have established an association between predicted VO_2 peak_ and both self-esteem and weight self-stigma, which were noted to explain the variance in VO_2 peak_ in OW women. Knowledge of this relationship will help in developing more efficient exercise programs that consider psychological factors. Public health Initiatives are required to implement coping strategies to enhance self-esteem and body satisfaction in OW individuals.

In our study, a lower predicted peak oxygen uptake was observed in the OW group, which corresponds to lower fitness levels. A strong relationship between FAC and obesity [39] and being overweight has been reported in multiple studies [13,40,41]. Our findings are consistent with a study by Pertovics et al. which demonstrated a negative association between being overweight and cardiorespiratory performance [12]. The present study sheds light on the internalization of weight-based stigmatization and self-esteem and its effect on fitness during treadmill exercise. Building on previous findings regarding self-esteem and being overweight, a study conducted among adolescents recognized the negative impact of a high body fat composition on self-esteem and depression, which aligned with our findings [42]. In parallel to the aforementioned study, the mediating effect of body acceptance on stigmatization and self-esteem has been established. One study showed that expressing body acceptance versus dissatisfaction by overweight individuals was associated with better self-esteem and less body-based stigmatization [17]. However, this finding is contradicted by another study that concluded that a high BMI did not influence self-esteem or depression [16]. These contrasting findings could result from differences in sociodemographic characteristics (e.g., age, race, and sex), as the focus of that study was socioeconomic status and its effect on psychological factors.

The prevalence of perceived weight self-stigma is a concerning issue, as it was high among OW participants in our study. Notably, our findings were consistent with those of a study by Murakami & Latner, which indicated that greater weight self-stigma was associated with lower self-esteem [16]. Greater weight self-stigma is known to have a potentially negative impact in both psychological and physiological contexts. A previous study reported that a greater weight stigma predicted a lower quality of life and higher psychological distress among women with a high-fat body composition [24]. The association between psychology and FAC is an area of interest in the literature. These changes in physical activity in relation to self-esteem have been shown to be mediated indirectly by self-efficacy and body image [43,44]. According to Sani et al., there is an indirect correlation between physical activity and self-esteem, mediated by the perceived body image of overweight individuals [44]. The negative perception of an individual’s body can lead to a decline in self-esteem, which can hinder participation in physical activities [44]. Preliminary findings are consistent with our results regarding the association between self-esteem, FAC, and exercise tolerance [45]. Aligned with our findings, it has been indicated that a high aerobic fitness is associated with higher self-esteem [45]. An exploratory study reiterated the strong detrimental influence of weight self-stigma on health behaviors [46]. A recent study has investigated the impact of weight stigma on physical activity and response to physical activity-related weight stigma. Results showed that in the context of physical activity, the women demonstrated a sense of shame, fear, sadness, and anger when they were judged based on their weight [47]. There are two plausible explanations for the effect of physical fitness on self-esteem: first, fitness is associated with a reduction in fat and an increase in lean tissue, which consequently enhances perceived body image and improves self-esteem [48]; second, from a physiological perspective, physical fitness enhances the release of neurochemicals in the brain, such as serotonin and endorphins, which positively affect mood [48]. The evidence from these studies suggests that psychological factors, such as weight self-stigma and self-esteem, may interplay with exercise capacity, and vice versa.

We are aware that our research may have some limitations. First, the cross-sectional design used in this study cannot conclude causal relationships between the variables. Future cohort studies are required to establish a cause-and-effect relationship and to support the findings in our study. Second, the fat mass percentage was measured indirectly using a bioimpedance (BIA) scale, which was used to estimate overall body composition; in comparison, the gold standard for evaluating body composition is dual-energy X-ray absorptiometry (DEXA). The BIA scale is effective for tracking changes in an individual’s body fat percentage changes over time [27]. Therefore, future studies with more direct densitometry and body composition measures, including magnetic resonance imaging and DEXA, are needed to confirm our findings. Third, the participants included only women between 18 and 45 years of age in Riyadh due to the high prevalence of women with obesity in Saudi Arabia [10]; therefore, the findings cannot be generalized to men or the subset of the population older than 45 years. Further studies should investigate the relationship between self-esteem, weight self-stigma, and FAC among men to examine the influence of gender-based differences in these relationships. Additionally, other physiological factors which were not investigated in this study may explain the variance in aerobic capacity. A potential factor that could explain this decline in oxygen uptake is the nature of muscle fibers. Multiple studies have observed that a larger proportion of type II muscle fibers are present in obese individuals [41,49]. Likely, differences in muscle fiber composition can substantially influence overall VO_2 peak_ [50]. The exact mechanisms underlying the decrease in oxygen consumption among OW individuals need to be elucidated upon in future research. Finally, the VO_2 peak_ was measured indirectly using the predictive VO_2 peak_ equation; however, the predictive equation has been validated and widely used in previous studies [29,51,52,53,54].

## 5. Conclusions

The findings of our study indicate that OW women have lower self-esteem, higher weight self-stigma, and lower FAC when compared with NW women. Furthermore, weight self-stigma and self-esteem were inversely associated and explained the variance in the FAC. Further investigations are needed to study the effects of weight loss intervention programs on psychological well-being and exercise tolerance among OW women. More broadly, research is needed to consider gender-based differences and other predictors that may explain the variance in FAC among OW individuals. Understanding the relationship between self-esteem, weight self-stigma, and FAC is necessary to guide healthcare providers and decision-makers to implement interventional models using cognitive behavioral therapy to improve self-esteem and weight stigmatization among OW women. Initiatives to promote physical activity should focus on helping women with obesity/overweight to establish coping strategies to reduce the burden of weight stigma. The results of this study are intended to orientate clinicians toward improving focus weight intervention to include cognitive behavioral therapy to enhance aerobic exercise capacity.

## Figures and Tables

**Table 1:** Baseline characteristics of the participants in the study.

Variables	Normal Weight *n* = 33	Overweight *n* = 33	*p*-Value
BMI (kg/m^2^)	20.4 ± 0.36	29.5 ± 0.8	<0.001 *
Height (cm)	161.6 ± 7.6	160.7 ± 5.0	0.56
Muscle mass (%)	70 ± 4	50 ± 30	<0.001 *
Fat mass (%)	30 ± 2	40 ± 3	<0.001 *
Age (Years)	21 ± 3	24 ± 7	0.01 *
Resting Systolic blood pressure (mmHg)	115 ± 13	121 ± 14	0.02 *
Resting Diastolic blood pressure (mmHg)	78 ± 8	82 ± 8	29
Resting heart rate (bpm)	89 ± 13	89 ± 12	0.9
Physical activity METS (min/week) ^a^	1405.3 ± 145.6	1537.7 ± 151.8	0.4

Data are presented as Mean ± SD. An independent *t*-test was used in the analysis. ^a^ measured using International Physical Activity Questionnaire (IPAQ); * *p* < 0.05 is considered significant.

**Table 2:** Comparison of mean exercise parameters, self-esteem, and weight self-stigma between groups.

Variables	Normal Weight *n* = 33	Overweight *n* = 33	*p*-Value
Exercise duration (min)	7.4 ± 1.1	6.7 ± 1.2	0.001 *
Heart rate peak (bpm)	179.7 ± 14.4	182.8 ± 8.3	0.13
Self-esteem ^a^	33 ± 3.7	30.6 ± 3.9	0.01 *
Bodyweight self-stigma ^b^	24.4 ± 6.9	32.4 ± 9.7	0.0003 *

Data are presented as Mean ± SD; ^a^ measured using a Rosenberg Self-Esteem Scale, a response that ranged between 10 and 40, where higher scores indicate higher self-esteem; ^b^ measured using a Weight Self-Stigma Questionnaire, the total score was the sum of all responses, varying from 12 to 60. A higher score indicates more shame related to body weight and shape; ANCOVA was used in the analysis; * *p* < 0.05 is considered significant.

**Table 3:** Multiple linear regression analyses to examine the independent associations between peak oxygen uptake and weight self-stigma and self-esteem.

Predictors	Coefficients						R^2^	VIF
	*β*	SE	*t*	*p*-Value	95% CI			
					Lower	Upper		
Constant	9.97	2.43	4.11	<0.001 *	5	14.9	0.45	
Age	0.06	0.02	2.42	0.02 *	0.008	0.11		2.4
BMI	−0.05	0.06	−0.87	0.40	−0.19	0.07		1.2
BodyFat %	−9.62	8.12	−1.18	0.24	−26.23	6.98		0.95
Weight self-stigma	−0.05	0.02	2.09	0.04 *	0.001	0.11		0.76
self-esteem	0.13	0.05	2.29	0.03 *	0.01	0.25		0.80
F (5, 61)	5.86							
*p*-value	0.001							

Model adjusted for Age, body mass index (BMI), Body Fat; * p < 0.05 is significant; Abbreviations: *β* = standardized beta; SE = standard error; CI = confidence interval; VIF = Variance Inflation Factor.

## Data Availability

The identified datasets analyzed during the current study are available from the corresponding author on reasonable request.
